# Electromyography as an intraoperative test to assess the quality of nerve anastomosis – experimental study on rats

**DOI:** 10.1515/med-2020-0143

**Published:** 2020-06-13

**Authors:** Norbert Czapla, Piotr Bargiel, Jan Petriczko, Daniel Kotrych, Piotr Krajewski, Piotr Prowans

**Affiliations:** Department of Plastic, Endocrine and General Surgery, Pomeranian Medical University, Szczecin, Poland; Department of Orthopaedics, Traumatology and Motor System Oncology, Pomeranian Medical University, Szczecin, Poland; Doctoral Programme at the Pomeranian Medical University in Szczecin, Żołnierska 54, 71-210, Szczecin, Poland

## Abstract

**Background:**

Many factors contribute to successful nerve reconstruction. The correct technique of anastomosis is one of the key elements that determine the final result of a surgery. The aim of this study is to examine how useful an electromyography (EMG) can be as an objective intraoperative anastomosis assessment method.

**Methods:**

The study material included 12 rats. Before the surgery, the function of the sciatic nerve was tested using hind paw prints. Then, both nerves were cut. The left nerve was sutured side-to-side, and the right nerve was sutured end-to-end. Intraoperative electromyography was performed. After 4 weeks, the rats were reassessed using the hind paw print analysis and electromyography.

**Results:**

An analysis of left and right hind paw prints did not reveal any significant differences between the length of the steps, the spread of the digits in the paws, or the deviation of a paw. The width of the steps also did not change.

Electromyography revealed that immediately after a nerve anastomosis (as well as 4 weeks after the surgery), better nerve conduction was observed through an end-to-end anastomosis. Four weeks after the surgery, better nerve conduction was seen distally to the end-to-end anastomosis.

**Conclusions:**

The results indicate that in acute nerve injuries intraoperative electromyography may be useful to obtain unbiased information on whether the nerve anastomosis has been performed correctly – for example, in limb replantation.

When assessing a nerve during a procedure, EMG should be first performed distally to the anastomosis (the part of the nerve leading to muscle fibers) and then proximally to the anastomosis (the proximal part of the nerve). Similar EMG results can be interpreted as a correct nerve anastomosis.

The function of the distal part of the nerve and the muscle remains intact if the neuromuscular transmission is sustained.

## 1 Introduction

Peripheral nerve injury with a loss of axonal continuity can be caused by major trauma or oncological resection. In such cases, the primary repair of a nerve gives the highest chance of regaining lost functions. The most common reconstruction method is an end-to-end anastomosis or, if tension-free repair is not possible, nerve grafting (usually with the sural nerve) [1]. Many factors determine the success of such nerve reconstruction. The most important reconstruction method is the correct anastomosis technique.

The decision to finish a nerve reconstruction is made when the anastomosis is accepted as technically correct. If considered faulty, it is repeated. Therefore, the only way to evaluate an anastomosis during an operation is by the surgeon’s judgment. Regardless of the clinical experience or quality of surgical loupes used, such assessment is always subjective. The authors of this article looked for another method to verify the visual intraoperative evaluation of nerve anastomosis.

The goal of this study is to examine how useful is EMG as an objective intraoperative nerve anastomosis assessment tool.

## 2 Materials and methods

Study material included 12 male Wistar rats weighing 250–300 g. Before surgery, sciatic nerve functions were evaluated by analyzing imprints of the hind paws. The hind paws were covered with ink, and animals were placed in a tunnel lined with paper. After covering a distance of 1 m, the length of steps and the geometry of their footprint were measured (the walking-track analysis) [2,3]. The animals were then anesthetized with a subcutaneous injection of ketamine hydrochloride at a dose of 120 mg/kg. After anesthesia, sciatic nerves were exposed on both limbs, and an EMG was performed. The nerves were cut, and a subsequent EMG was performed to confirm the loss of nerve conduction. The left sciatic nerve was sutured side-to-side, essentially preventing nerve bundles from contacting. This was to simulate a faulty anastomosis. The right sciatic nerve was sutured end-to-end, paying attention to the correct adaptation of the ends, ensuring the proper contact of nerve bundles. The anastomosis was performed by applying four 10/0 stitches with the use of surgical loupes magnifying the field 4.5 times. Afterward, another EMG examination was performed to assess the conduction through the anastomoses. Surgical wounds were closed with 4/0 skin sutures. Following the surgery, animals received standard food and drinking water without restrictions. After 4 weeks, the footprints were again sampled over the distance of 1 m. Then (after anesthesia) sciatic nerves were exposed, revealing the site of anastomosis. A nerve conduction test was performed.

**Figure 1:**
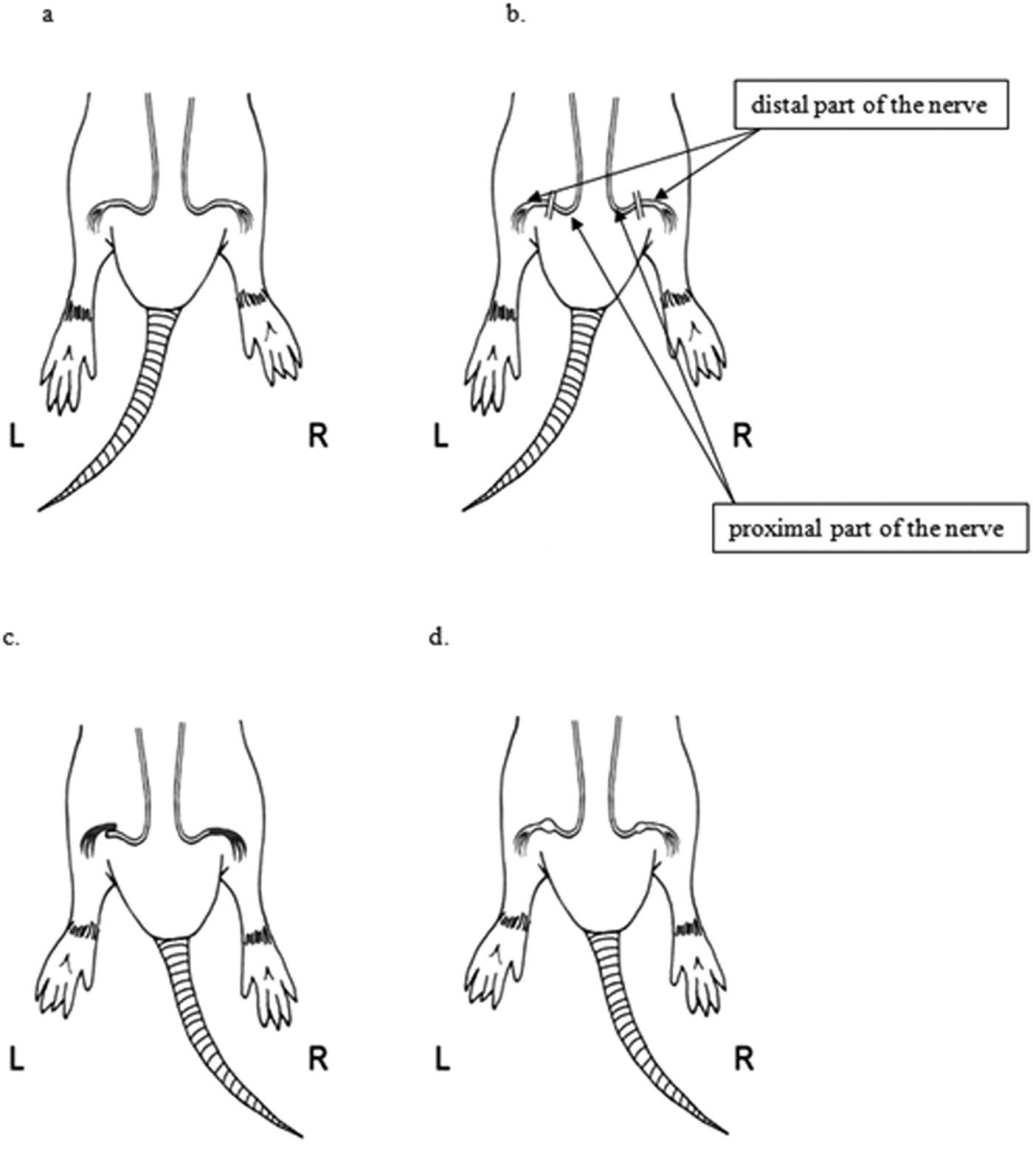
The schematic representation of (a) exposure of sciatic nerves, (b) transecting the nerves, (c) anastomosis L (left side) side-to-side, R (right side) end-to-end, and (d) follow-up after 4 weeks.

**Figure 2:**
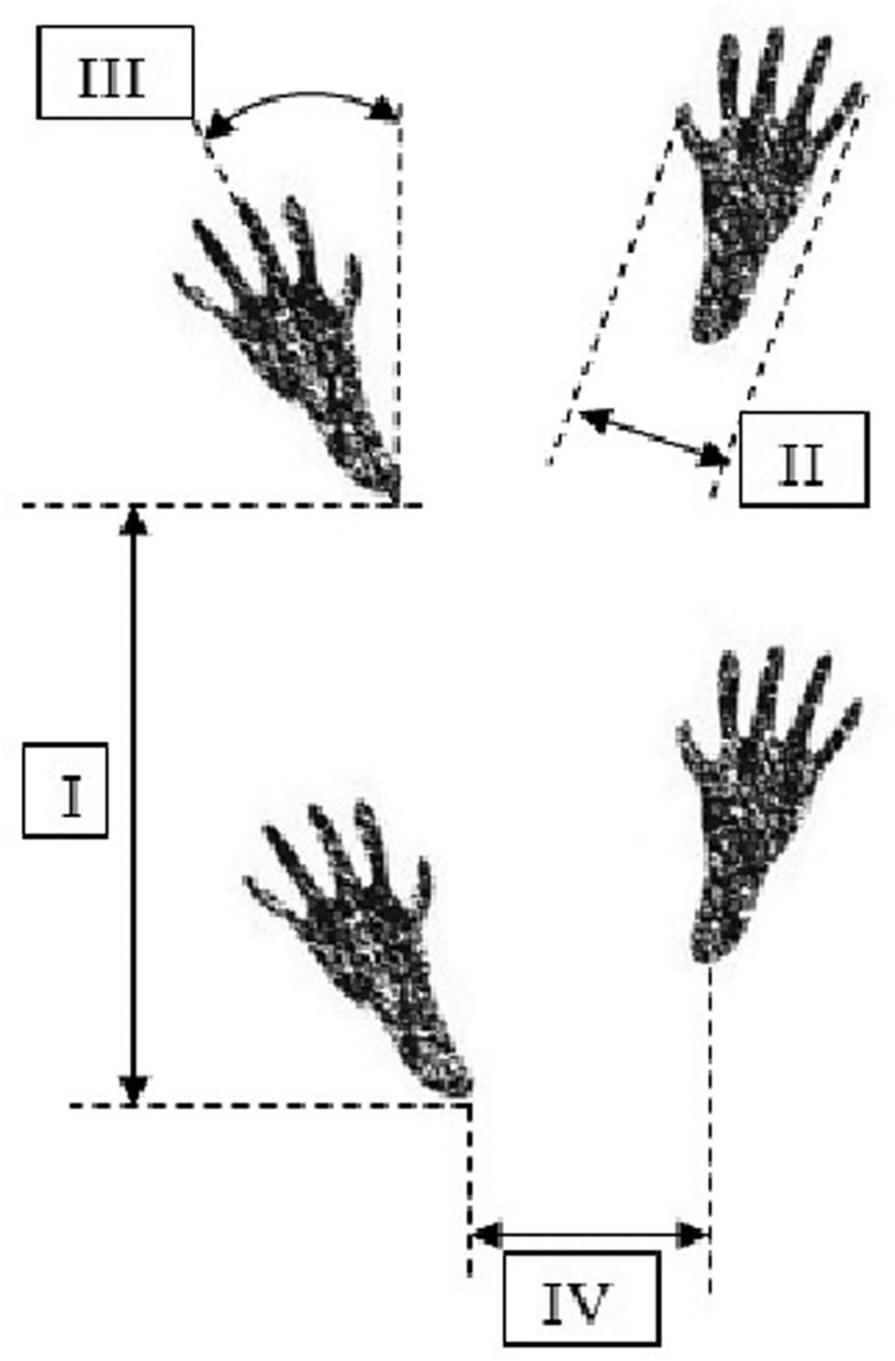
The a schematic representation of gait assessment based on the measurements of the hind paw prints: (I) step length, (II) digit spread (between digits I–V), (III) foot deviation (angle between the foot axis and the walking axis), and (IV) step width (distance between the left heel and the right heel).

Nerve anastomoses were sampled for microscopic examination. Shortly thereafter, the animals were euthanized with an injection of pentobarbital sodium. EMG was performed using a NerveMonitor C2 (inomed Medizintechnik GmbH, Germany). The nerves were stimulated at 2 mA. Tissues for microscopic examination were stained with hematoxylin and eosin. The presence of nerve bundles in the anastomosis was assessed. The T-test for dependent samples was used for statistical calculations. The Local Ethics Committee approval number 7/2015 was obtained for conducting experiments on animals. The animals showed no signs of suffering in the postoperative period; therefore, there was no need for additional pain management. Figures 1 and 2 schematically present surgical technique and the method of measuring gait parameters based on the paw prints, respectively.

## 3 Results

Individual gait parameters as an indirect measurement of the sciatic nerve function are presented in Tables 1 and 2.

**Table 1:** The length of the steps (distance between successive footprints on the right and left side [mm]), the spread of the digits (distance between digits I and V [mm]), and the deviation of the foot (angle between the axis of the foot and the axis of the body [°])

No.	Step parameters	Left		Right	
		Before cutting	Follow-up after 4 weeks	Before cutting	Follow-up after 4 weeks
1	Step length [mm]	68	101	68	117
	digit spread [mm]	23	12	24	11
	Deviation of the foot [°]	32	19	27	32

2	Step length [mm]	105	85	120	100
	Digit spread [mm]	23	12	23	11
	Deviation of the foot [°]	16	23	15	19

3	Step length [mm]	145	112	135	105
	Digit spread [mm]	24	12	24	13
	Deviation of the foot [°]	15	20	20	20

4	Step length [mm]	132	120	145	107
	Digit spread [mm]	20	10	21	14
	Deviation of the foot [°]	17	20	29	24

5	Step length [mm]	143	145	147	120
	Digit spread [mm]	21	13	21	11
	Deviation of the foot [°]	22	26	27	25

6	Step length [mm]	160	120	144	120
	Digit spread [mm]	24	11	24	16
	Deviation of the foot [°]	27	24	26	29

7	Step length [mm]	160	93	150	90
	Digit spread [mm]	23	13	23	15
	Deviation of the foot [°]	9	17	9	13

8	Step length [mm]	140	123	140	120
	Digit spread [mm]	21	10	20	10
	Deviation of the foot [°]	24	11	19	23

9	Step length [mm]	120	115	120	117
	Digit spread [mm]	20	10	20	10
	Deviation of the foot [°]	23	22	23	14

10	Step length [mm]	150	113	153	115
	Digit spread [mm]	23	13	23	11
	Deviation of the foot [°]	23	16	24	18

11	Step length [mm]	120	110	120	112
	Digit spread [mm]	22	12	24	12
	Deviation of the foot [°]	20	10	22	14

12	Step length [mm]	118	109	120	107
	Digit spread [mm]	24	12	24	12
	Deviation of the foot [°]	12	12	13	13

Average	Step length [mm]	130.08	112.16	130.16	110.83
	Digit spread [mm]	22.33	11.66	22.58	12.16
	Deviation of the foot [°]	20.00	18.33	21.16	20.33
Standard deviation	Step length [mm]	26.13	15.29	23.36	9.35
	Digit spread [mm]	1.49	1.15	1.62	1.94
	Deviation of the foot [°]	6.50	5.24	6.20	6.38

**Table 2:** The width of the steps (the distance between the hind paws) before the transection of the nerves and 4 weeks after reconstruction

No.	Step width [mm]	
	Before transection	Follow-up after 4 weeks
1	54	40
2	55	65
3	35	36
4	52	55
5	35	35
6	39	25
7	42	48
8	36	51
9	50	34
10	45	60
11	47	55
12	50	60
Average	45.00	47.00
Standard deviation	7.44	12.69

The length of the steps was statistically shorter (*p* = 0.03) 4 weeks after the side-to-side anastomosis was performed, compared to the length before the nerve was cut. Digit spread was statistically smaller (curled-up foot; *p* < 0.01). However, there were no statistical differences in the deviation of the foot from the walking axis.

The length of steps 4 weeks after the end-to-end anastomosis was statistically shorter (*p* = 0.03) compared to the length of the step before the nerve was cut. Digit spread was statistically smaller (curled up foot; *p* = 0.01). There was no difference in the deviation of the foot from the walking axis.

The width of the steps (the distance between the hind paws) did not change 4 weeks after the nerve reconstruction.

Statistical analysis showed that the function of the sciatic nerve, expressed in the ability to walk, was statistically worse, regardless of the manner of the anastomosis. The comparison of walking imbalances 4 weeks after the nerve anastomosis did not reveal any statistically significant differences between side-to-side and end-to-end anastomosis in regards to the length of steps, digit spread, and foot deviation.

The measurements obtained in the electrophysiological examination of sciatic nerves are presented in Tables 3 and 4.

**Table 3:** Electrophysiological parameters upon stimulating the sciatic nerves shortly after the surgical anastomosis.

Lp.	Stimulation response	Directly after the nerve anastomosis			
		Left sciatic nerve, side-to-side anastomosis		Right sciatic nerve, end-to-end anastomosis	
		The proximal part of the nerve	The distal part of the nerve	The proximal part of the nerve	The distal part of the nerve
1	Voltage [mV]	0	6.39	6.51	6.5
	Time [ms]	0	1.35	1.05	1.05
2	Voltage [mV]	0	6.48	6.57	6.58
	Time [ms]	0	1.45	1.2	1.2
3	Voltage [mV]	0	6.5	6.48	6.5
	Time [ms]	0	1.35	1.2	1.2
4	Voltage [mV]	0	6.46	0	1.63
	Time [ms]	0	1.15	0	1.3
5	Voltage [mV]	0	5.91	5.83	5.81
	Time [ms]	0	1.1	1.4	1
6	Voltage [mV]	0	6.58	6.48	6.58
	Time [ms]	0	1.2	1.8	1.1
7	Voltage [mV]	0	6.42	6.58	6.58
	Time [ms]	0	1.7	1.9	1.15
8	Voltage [mV]	0	0.72	5.93	5.9
	Time [ms]	0	1.4	1.05	1.1
9	Voltage [mV]	0.11	6.5	6.45	6.37
	Time [ms]	6.95	1.1	1.15	2.6
10	Voltage [mV]	0	3.6	6.52	6.52
	Time [ms]	0	1.95	1.15	1.2
11	Voltage [mV]	0	4.51	5.33	4.62
	Time [ms]	0	1.25	1.1	1.45
12	Voltage [mV]	0	4.3	6.3	3.52
	Time [ms]	0	1.35	1.05	1.05
Average	Voltage [mV]	0.0092	5.3642	5.7483	5.5925
	Time [ms]	0.5791	1.3625	1.1708	1.2833
Standard deviation	Voltage [mV]	0.0317	1.7968	1.8505	1.5702
	Time [ms]	2.0063	0.2506	0.4673	0.4323

**Table 4:** The electrophysiological study parameters conducted after stimulating the sciatic nerves 4 weeks after the nerve anastomosis was performed

No.	Stimulation response	Follow-up after 4 weeks			
		Left sciatic nerve, side-to-side anastomosis		Right sciatic nerve, end-to-end anastomosis	
		The proximal part of the nerve	The distal part of the nerve	The proximal part of the nerve	The distal part of the nerve
1	Voltage [mV]	0	0	0.41	0.31
	Time [ms]	0	0	5.25	3.86
2	Voltage [mV]	0	0	0.17	0.19
	Time [ms]	0	0	5.65	4.75
3	Voltage [mV]	0	0	0.09	0.09
	Time [ms]	0	0	6.2	5.7
4	Voltage [mV]	0	0	0.11	0
	Time [ms]	0	0	14.3	0
5	Voltage [mV]	0.11	0.06	0	0
	Time [ms]	9.06	8.35	0	0
6	Voltage [mV]	0	0	1.13	1.19
	Time [ms]	0	0	3.2	5.8
7	Voltage [mV]	0	0	0.32	0.26
	Time [ms]	0	0	7.6	7.75
8	Voltage [mV]	0	0	0	0
	Time [ms]	0	0	0	0
9	Voltage [mV]	0	0.03	0.5	0.4
	Time [ms]	0	6.15	12.45	12.15
10	Voltage [mV]	0	0	0.33	0.34
	Time [ms]	0	0	9.6	12.65
11	Voltage [mV]	0.2	0	0.08	0.13
	Time [ms]	1.85	0	1.15	2.65
12	Voltage [mV]	0	0	0.05	0.04
	Time [ms]	0	0	3.2	3.2
Average	Voltage [mV]	0.0258	0.0075	0.9091	1.2083
	Time [ms]	0.2658	0.2458	5.7166	4.6414
Standard deviation	Voltage [mV]	0.0633	0.0186	2.6213	2.8607
	Time [ms]	0.3185	0.3293	4.8758	4.2988

The values of potentials registered in newly made side-to-side anastomoses were statistically lower (*p* < 0.001) in comparison to a healthy nerve. Zero conduction time is understood as a complete absence of conduction in a side-to-side anastomosis.

Neither the values of the potentials registered passing through the newly created end-to-end anastomoses nor the conduction time showed differences in comparison to a healthy nerve. The result indicates that a correctly performed anastomosis of a freshly damaged nerve ensures proper conduction of the impulse to the muscle.

The potentials registered passing through newly created anastomoses were higher in the end-to-end anastomoses than that in the side-to-side anastomoses (*p* < 0.001). However, there were no statistical differences in conduction time. These results indicate that an end-to-end anastomosis (i.e., a technically correct nerve anastomosis) provides better conductivity.

An analysis of potentials registered on stimulating the distal part of the nerve 4 weeks after performing the anastomosis revealed that in end-to-end anastomoses, potentials were higher (*p* = 0.02). In contrast, the conduction time through end-to-end anastomoses was longer (*p* = 0.02). This means that the distal part of the nerve and the muscles beyond a side-to-side anastomosis did not regain function.

Moreover, there was a significant difference (*p* = 0.02) when comparing the potentials registered on stimulating the proximal part of the nerve in side-to-side and end-to-end anastomoses. The potentials registered in side-to-side anastomoses were higher. The conduction time in the end-to-end anastomosis was longer (*p* = 0.049), which means that side-to-side anastomoses did not conduct any impulses.

An analysis of a correlation between gait parameters and the results of electrophysiological studies did not reveal statistical differences, meaning that the function of both limbs was impaired, regardless of the anastomosis technique used.

**Figure 3:**
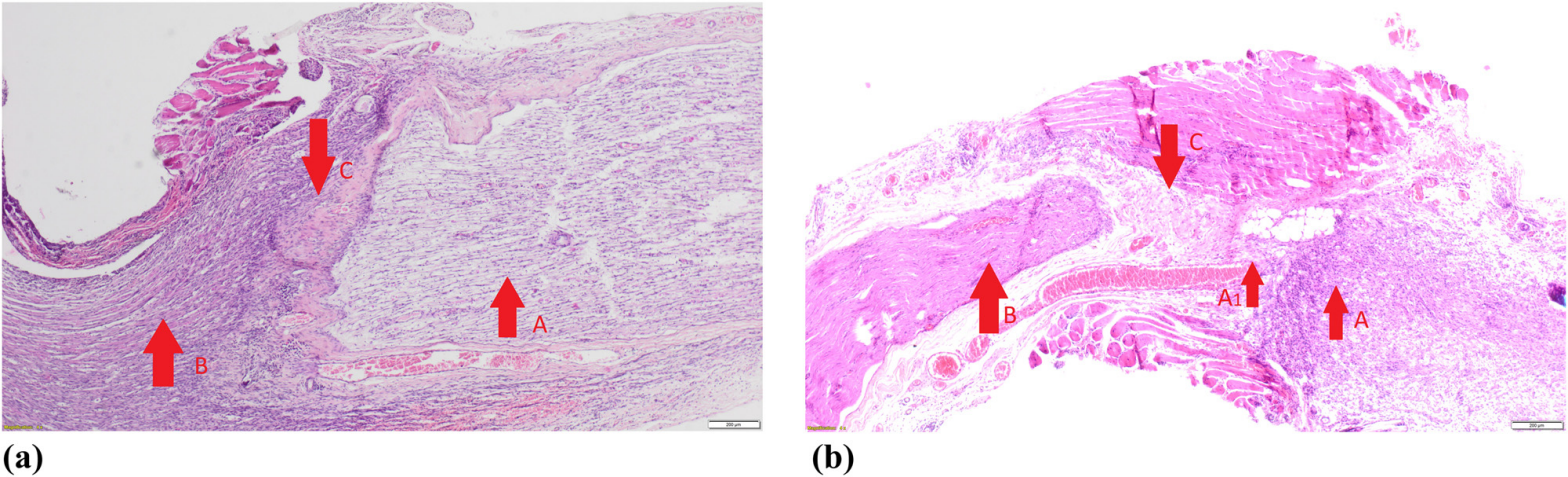
Pathomorphological examples of a side-to-side (a) and an end-to-end (b) nerve anastomoses.

The morphological analysis of nerve anastomoses at 400× magnification showed nerve fibers both of the proximal (arrow B) and distal sciatic nerve stumps (arrow A) with a thick layer of connective scar tissue in the middle (arrow C) in Figure 3a, which was confirmed to be blocking nerve conduction in electrophysiological examinations. Evaluation of an end-to-end anastomosis (Figure 3b) showed loose connective tissue (arrow C) and single nerve endings passing through the scar (arrow A1) between the proximal and distal ends of the sciatic nerve (arrows A and B), which corresponds with a positive response on stimulation in EMG.

## 4 Discussion

In side-to-side anastomoses, nerve stimulation on the proximal end of the anastomosis after 4 weeks did not show conduction in ten cases. These results seem to confirm that a faulty anastomosis, in which the bundles of the nerve endings are not in contact with each other, dramatically lowers the chance of nerve function return. In two cases, a follow-up EMG after 4 weeks showed nerve conduction. This phenomenon was described previously by other authors who described the ingrowth of nerve fibers in the end-to-side [2,4,5,6,7,8,9] or the side-to-side anastomosis [3,10]. In the aforementioned studies, a nerve sheath was cut at the site before the anastomosis was made to ensure direct contact of nerve fibers between the end and the side of the fused nerves. This maneuver was supposed to increase the likelihood of reinnervation after nerve reconstruction. In our study, the sides of the fused nerves still had their sheath on. It is possible that suturing created micro-openings in the sheath, enabling nerve fibers to grow in and eventually allowing nerve function return.

In nerves stimulated 4 weeks after an end-to-end anastomosis, nerve conduction was achieved in ten cases. Such results are consistent with the literature, confirming that good reinnervation depends on the correct anastomosis technique.

After 4 weeks, stimulation of the proximal and distal end of a side-to-side anastomosis showed muscle response in two cases. Based on this result, one could assume that a lack of muscle response could be either due to the lack of conduction in the anastomosis or degenerative changes in the denervated muscle. However, when analyzing the end-to-end nerve conduction results (in which muscular response was obtained after the proximal end stimulation), it seems that the primary reason for the lack of muscle response after side-to-side anastomoses is the lack of conductivity in these anastomoses. Muscle degeneration is secondary.

Interestingly, performing EMG on freshly anastomosed nerves demonstrated that the nerve sutured end-to-end conducted electric impulses in 12 cases, while the nerve sutured side-to-side only in one case. Conduction of impulses in a reconstructed nerve was ensured by the direct contact of nerve bundles. In end-to-end anastomoses, this result seems obvious. However, in side-to-side anastomoses, nerve bundles were not in contact. One case of conduction can probably be explained by direct contact of nerve fibers resulting from the micro-damage of the sheath caused by the suturing. Conduction through an end-to-end anastomosis also correlated with later nerve function. It seems, therefore, that intraoperative EMG examination can be used to confirm the correct approximation and alignment of nerve bundles in anastomoses. Literature describes the use of EMG for late evaluation of nerve regeneration [4,9,11,12,13,14].

In two subjects with an end-to-end anastomosis, a follow-up EMG proximal to the anastomosis after 4 weeks did not show nerve conduction. In one of these cases, nerve anastomosis separation was observed during the surgical reevaluation of the nerve. It could have been caused by the motor activity of the animal causing repetitive stress on the anastomosis. Similar complications are observed in clinical practice in patients who do not adhere to postoperative guidelines of rest and limb immobilization after surgery.

When interpreting the results of this experiment and similar functional disorders of both limbs regardless of the technique used for nerve anastomosis, it can be assumed that the amount of time required to restore the function of a damaged nerve leads to degenerative changes in muscles it innervates. Thus, regardless of a correctly performed nerve anastomosis, the excessive time needed for nerve regeneration causes changes in the muscles and leads to poor results. Electrophysiological examination showed better nerve function after an end-to-end anastomosis. However, the final functional outcome also depends on another component, that is, degenerative changes in the muscles. This is confirmed by clinical observations with significantly worse results of motor or mixed nerve reconstructions if done more than 1 year after the injury [15,16].

## 5 Conclusions

The results indicate that in acute nerve injuries intraoperative electromyography may be useful to obtain unbiased information on whether the nerve anastomosis has been performed correctly – for example, in limb replantation.

When assessing a nerve during a procedure, EMG should be first performed distally to the anastomosis (the part of the nerve leading to muscle fibers) and then proximally to the anastomosis (the proximal part of the nerve). Similar EMG results can be interpreted as a correct nerve anastomosis.

The function of the distal part of the nerve and of the muscle remains intact if the neuromuscular transmission is sustained.
